# Evaluation of a Novel Tc-99m Labelled Vitamin B_12_ Derivative for Targeting *Escherichia coli* and *Staphylococcus aureus In Vitro* and in an Experimental Foreign-Body Infection Model

**DOI:** 10.1007/s11307-015-0832-x

**Published:** 2015-04-10

**Authors:** Daniela Baldoni, Robert Waibel, Peter Bläuenstein, Filippo Galli, Violetta Iodice, Alberto Signore, Roger Schibli, Andrej Trampuz

**Affiliations:** Infectious Diseases Research Laboratory, Department of Biomedicine, University Hospital, Basel, Switzerland; Center for Radiopharmaceutical Science, Paul Scherrer Institute, Villigen PSI, Switzerland; Nuclear Medicine, Department of Medical-Surgical Sciences and of Translational Medicine, Faculty of Medicine and Psychology, “Sapienza” University, Ospedale S. Andrea, via di Grottarossa 1035, 00189 Rome, Italy; Septic Surgery Unit, Center for Musculoskeletal Surgery, Charité - University of Medicine, Berlin, Germany

**Keywords:** Vitamin B_12_, ^99m^Tc, *Escherichia coli*, *Staphylococcus aureus*, Infection imaging

## Abstract

**Purpose:**

Vitamin B_12_ (cyanocobalamin, Cbl) is accumulated by rapidly replicating prokaryotic and eukaryotic cells. We investigated the potential of a Tc-99m labelled Cbl derivative ([^99m^Tc]PAMA(4)-Cbl) for targeting infections caused by *Escherichia coli* and *Staphylococcus aureus. In vitro* binding assays were followed by biodistribution studies in a mouse model of foreign body infection.

**Procedures:**

*E. coli* (ATCC 25922) and *S. aureus* (ATCC 43335) were used as test strains. [^57^Co]Cbl, [^67^Ga]citrate and [^99m^Tc]DTPA served as reference compounds. The *in vitro* competitive binding of [^57^Co]Cbl or [^99m^Tc]PAMA(4)-Cbl, and unlabeled Cbl, to viable or killed bacteria, was evaluated at 37 and 4 °C. A cage mouse model of infection was used for biodistribution of intravenous [^57^Co]Cbl and [^99m^Tc]PAMA(4)-Cbl in cage and dissected tissues of infected and non-infected mice.

**Results:**

Maximum binding (mean ± SD) of [^57^Co]Cbl to viable *E. coli* was 81.7 ± 2.6 % and to *S. aureus* 34.0 ± 6.7 %, at 37 °C; no binding occurred to heat-killed bacteria. Binding to both test strains was displaced by 100- to 1000-fold excess of unlabeled Cbl. The *in vitro* binding of [^99m^Tc]PAMA(4)-Cbl was 100-fold and 3-fold lower than the one of [^57^Co]Cbl for *E. coli* and *S. aureus*, respectively. *In vivo*, [^99m^Tc]PAMA(4)-Cbl showed peak percentage of injected dose (% ID) values between 1.33 and 2.3, at 30 min post-injection (p.i.). Significantly higher retention occurred in cage fluids infected with *S. aureus* at 4 h and with *E. coli* at 8 h p.i. than in non-infected animals. Accumulation into infected cages was also higher than the one of [^99m^Tc]DTPA, which showed similar biodistribution in infected and sterile mice. [^57^Co]Cbl gradually accumulated in cages with peaks % ID between 3.58 and 4.83 % achieved from 24 to 48 h. Discrimination for infection occurred only in *E. coli*-infected mice, at 72 h p.i. [^67^Ga]citrate, which showed a gradual accumulation into cage fluids during 12 h, was discriminative for infection from 48 to 72 h p.i. (*P* < 0.05).

**Conclusion:**

Cbl displayed rapid and specific *in vitro* binding to test strains. [^99m^Tc]PAMA(4)-Cbl was rapidly cleared from most tissues and discriminated between sterile and infected cages, being a promising candidate for imaging infections in humans.

## Introduction

Bacterial infections are an important cause of morbidity and mortality worldwide. The accurate diagnosis of infection (or its exclusion) is the first crucial step in the management of these patients. Imaging techniques constitute a non-invasive and attractive approach that, in the last decades, has gained on importance by combining visualization of radiopharmaceuticals and morphological imaging, with positron emission tomography/X-ray computed tomography (CT) and single photon emission computed tomography/CT imaging [1, 2]. In various types of infections, including prosthetic joint infections, radionuclide imaging techniques become essential when the diagnosis remains unclear [3]. The current standard method is based on labelling white blood cells isolated from patients [4–7]. Other agents have been developed in the past decade, such as radiolabeled antimicrobials (ciprofloxacin, sparfloxacin, ceftizoxime, isoniazid, ethambutol, fluconazole), antimicrobial peptides (29–31 UBI, human beta-defensin-3), cytokines (IL-8), 2-deoxy-2-[^18^F]fluoro-D-glucose, growth factors and bacteriophages [2, 8–15]. However, in pre-clinical and clinical studies, these agents showed limitations, in particular insufficient specificity for diagnosis infection, making them unsuitable in the clinical practice [1, 16, 17].

Vitamin B_12_ or cobalamin (Cbl) is an important hydrophilic enzyme cofactor required in fast replicating cells, such as bacteria or fungi. In bacteria, Cbl catalyses transmethylation and rearrangement reactions by binding to Cbl-dependent enzyme isoforms, directly or indirectly responsible for the synthesis of ATP, amino acids and nucleotides [18]. Comparative genomic analysis revealed a wide distribution of genetic elements involved in the regulation or uptake of Cbl derivatives [18]. Cbl transport systems were studied mainly in enteric bacteria. Especially, the *Escherichia coli* Cbl uptake is mediated by an external membrane transporter (BtuB, TonB-dependent), which transfer Cbl to a periplasmatic protein (BtuF) and finally across the inner membrane (BtuCD, ABC ATP-dependent) [19–21]. Gram-positive bacteria, as *Staphylococcus aureus*, do not have an outer membrane, and thus completely lack the BtuB carrier. However, element analogues to the *E. coli* BtuCD inner membrane transporter were reported in *S. aureus* and *Staphylococcus epidermidis* strains, and described as less specific carriers of Cbl, and closely related molecules as heme and siderophores [18]. The latter findings supported previous *in vitro* studies of [^57^Co]Cbl uptake mechanism, which reported high avidity of binding to several pathogen bacteria in different culture conditions [22, 23].

The distinctive uptake mechanism in bacterial and animal cells may represent the basis for development of radiolabeled Cbl derivatives for specific targeting of bacterial infections and for reduced systemic accumulation *in vivo*. Cbl distributes through the blood circulation upon binding to the transport protein transcobalamin II (TC II). The Cbl-TC II complex is rapidly internalized through binding to the transcobalamin II receptor (RTC II) and megalin receptor, mainly expressed in humans in the kidneys, liver, intestine lumen, glands and absorptive epithelia [24, 25]. A second family of proteins, transcobalamin I (TC I or haptocorrins, or R-type Cbl binders), binds free Cbl and cobinamides in the blood. The TC I protein family was mainly found in secondary granules of granulocytes and in salivary glands, and its release has a protective function of reducing pathogen colonization and growth [26, 27].

A [^57^Co]Cbl oral formulation was initially developed and used in humans for diagnosis of vitamin B_12_ malabsorption syndromes [28]. A similar intravenous formulation showed potential targeting of tumour cells [29]. However, the long half-life of the isotope [^57^Co]Cbl, together with its systemic non-specific accumulation and persistence in several organs, limited the maximal injectable dose to 1 μCi. This dose limit prohibits the use of [^57^Co]Cbl for imaging purposes. Therefore, vitamin B_12_ analogues were synthesized and labelled with isotopes such as In-111 and Tc-99m. An In-111 labelled Cbl derivative, diethylenetriamine-pentaacetate adenosylcobalamin ([^111^In]DAC), showed promising results for the diagnosis of various malignancies. Interestingly, in the same study, [^111^In]DAC derivative accumulated in the right wrist of one patient, who was diagnosed a staphylococcal septic arthritis [30]. However, the high unspecific accumulation of the [^111^In]DAC in non-target tissues made this radiopharmaceutical inappropriate for routine clinical application.

**Fig. 1.**
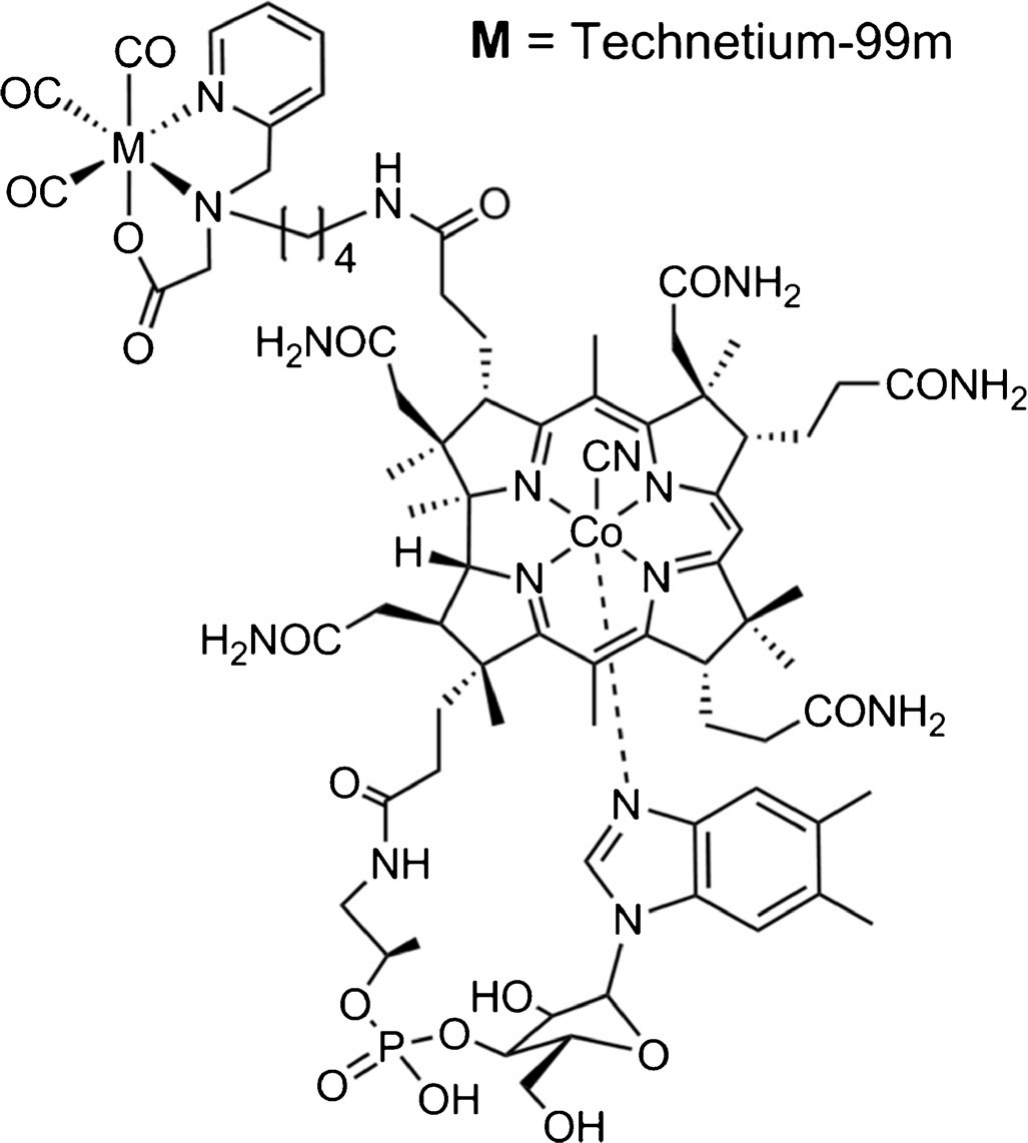
Chemical structure of [^99m^Tc]PAMA4-Cbl.

The Cbl derivative [^99m^Tc]PAMA(4)-Cbl has been recently developed and tested for imaging of malignancies (Fig. 1) [31, 32]. This conjugate carries mono-anionic ligands with a NNO donor set and can be efficiently labelled with [[^99m^Tc](OH_2_)_3_(CO)_3_]^+^ at yields >95 % under mild conditions (50 °C, 60 min) [31]. Importantly, [^99m^Tc]PAMA(4)-Cbl has abolished binding to the major Cbl transport protein TC II. As a consequence, significantly lower uptake in non-targeted tissue and organs was demonstrated in mice bearing different tumour types.

**Fig. 2.**
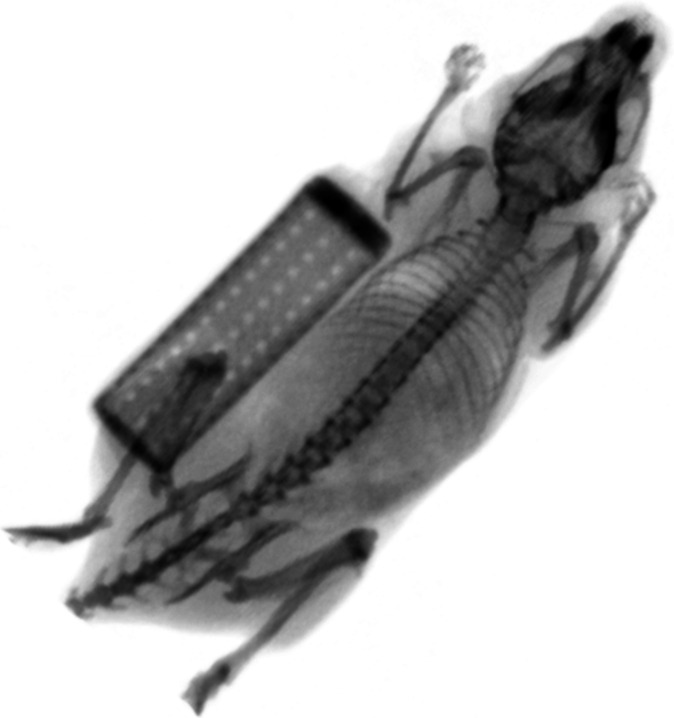
CT scan of a C57Bl/6 mouse with subcutaneous implanted tissue cage, 2 weeks after surgery.

In this study, we evaluated the potential of [^99m^Tc]PAMA(4)-Cbl for specific targeting *E. coli* and *S. aureus in vitro* and in a cage model of foreign body infection in mice (Fig. 2) [33]. The results were compared with the TC II binding molecule [^57^Co]Cbl. The infection model of subcutaneously implanted cages has been used in mice for investigating pathophysiology and treatment efficacy of implant-associated infection [34–36]. The kinetics and histology of the infected cage closely resemble a human infection of prosthesis with bacteria adherent to the foreign body, infiltration of granulocytes and pus. The model allows the induction of a localized persistent infection, characterized by a high and reproducible bacterial density of 10^7^–10^9^ colony forming units (CFU) per millilitre of cage fluid. Cages fill with inflammatory fluid (exudate) surrounded by a highly vascularized tissue. We performed biodistribution studies with [^99m^Tc]DTPA to evaluate the local vascularization of infected and non-infected cages. In addition, we tested the performance of [^67^Ga]citrate, an agent accumulating unspecifically at sites of aseptic inflammation and infection, for targeting tissue cage infections.

## Materials and Methods

### Radiopharmaceuticals

[^99m^Tc]PAMA(4)-cyanocobalamin ([^99m^Tc]PAMA(4)-Cbl) was synthesized and labelled as described elsewhere [32]. Five gigabecquerels of sodium [^99m^Tc]pertechnetate was added to the kit and heated for 20 min. The alkaline solution was neutralized with a 1-M HCl solution and additionally buffered with 1 M MES. This solution was added to 30 μg of the lyophilized Cbl-b-(butyl)-PAMA-OEt. The reaction mixture was kept at 75 °C for 75 min. The product was purified over a RP-8 column (X-Terra RP8 5 μm 30 × 150 mm) using a gradient of solvent A (10 % ethanol, 90 % 0.1 M acetate buffer) and B (70 % ethanol/water). The collected fraction of about 1 ml was diluted with phosphate buffer at pH 7.4 up to 10 ml. Aliquots of the product were distributed according to the needs. Briefly, DTPA (Pentacis®, IBA Molecular, Switzerland) was labelled in 10 ml of sterile 0.9 % saline with 3.05 GBq of sodium [^99m^Tc]pertechnetate, according to the manufacturer’s instructions, with a labelling efficiency of 98.6 %. [^57^Co]cyanocobalamin ([^57^Co]Cbl) 0.39 MBq/50 ng/ml was purchased by MP Biomedicals (Diagnostic Division, Orangeburg, NY, USA). Gallium-67 citrate solution for injection was purchased at a radiochemical concentration of 17.17 MBq/ml (Mallinckodt Schweiz AG, Radiopharma, Wollerau, Switzerland).

### Test Microorganisms

Laboratory strains *E. coli* (ATCC 25922) and *S. aureus* (ATCC 35556, methicillin susceptible) were used. Bacteria were stored at −70 °C using a cryovial bead preservation system (Microbank, Pro-Lab Diagnostics, Richmond Hill, ON, Canada). Single cryovial beads were cultured overnight on Columbia sheep blood agar plates (Becton Dickinson, Heidelberg, Germany).

For *in vitro* binding studies, overnight cultures were prepared in snap-lid tubes from 2 to 3 CFU in 5 ml of a synthetic minimal medium depleted of vitamin B_12_. After 18–20 h of incubation at 37 °C and 200 rpm, the cultures were diluted 1:100 in the same medium and further incubated at 37 °C to mid-logarithmic phase in sealed tubes.

For *in vivo* studies, overnight cultures were prepared in 5 ml tryptic soy broth (TSB), incubated at 37 °C for 18–20 h. Bacterial suspensions were then washed three times, resuspended in 5 ml of sterile 0.9 % saline and diluted.

### *In Vitro* Binding Studies

The *in vitro* binding and internalization profiles of [^57^Co]Cbl and [^99m^Tc]PAMA(4)-Cbl to *E. coli* and *S. aureus* were characterized. Stock solutions of the radiotracers were prepared at a concentration of 0.004 MBq/ml (0.350–0.700 ng/ml, 0.3–0.5 pmol/ml) for [^57^Co]Cbl and ≈1 MBq/ml (≈0.5 ng/ml, ≈0.3 pmol/ml) for [^99m^Tc]PAMA(4)-Cbl in phosphate-buffered solution (PBS). In competition studies, 10-fold dilutions between 3 and 0.003 μg/ml of unlabeled Cbl (Sigma-Aldrich, Steinheim, Germany) were added to the stock radiotracer solutions.

Bacterial cultures in the logarithmic phase at OD_600_ between 0.4 and 0.6 were centrifuged and resuspended in equal volume of sterile PBS. Five hundred microlitres of resuspended cultures were transferred to Eppendorf tubes and used for the measurement of binding to viable bacteria at 37 °C. For testing binding at 4 °C, bacterial suspension in PBS were let to equilibrate for 1 h at 4 °C before adding the radiopharmaceuticals. In order to evaluate the binding to killed bacteria, bacterial cultures were either exposed to heat at 99 °C or to 70 % ethanol at 4 °C for 30 min (*E. coli*) or 60 min (*S. aureus*). The bacterial suspensions were then centrifuged, resuspended in PBS and used for further studies. The PBS resuspended heat-killed or ethanol-killed bacteria were sampled on Columbia blood agar plates, and the CFU were enumerated after 24 h of incubation at 37 °C. Colony counts remained <10 CFU/ml.

Five hundred microlitres of each radiopharmaceutical solution was added to the 500 μl of bacterial suspensions. Binding assays were performed in quadruplicate vials per testing conditions. Afterwards, vials were centrifuged for 5 min at 13,500 rpm and 4 °C, and pellets were washed with 500 μl of cooled PBS. Supernatants and resuspended pellets were counted in a multi-well NaI γ-counter (Cobra; Packard, USA) and the counts per minute (CPM) recorded. The percentage of radiolabeled Cbl in the pellets was calculated as the percentage of the CPM_p_/CPM_0_ ratio per log_10_ 8.0 CFU/ml, where CPM_p_ were the CPM associated to pellets and CPM_0_ were the total CPM of the radiolabeled Cbl added per vial.

*In vitro* binding of the radiotracers was measured after 5, 30, 60, 120 min (*E. coli* and *S. aureus*) and 180 min (*S. aureus*) of incubation at 37 and 4 °C.

Competition binding studies were measured for cultures incubated for 1 h (*E. coli*) and 3 h (*S. aureus*), at 37 and 4 °C, simultaneously with 10-fold serial dilution of unlabeled Cbl and either ^[57^Co]Cbl or [^99m^Tc]PAMA(4)-Cbl. In addition, competition with unlabeled Cbl was studied on bacterial cultures previously incubated with [^57^Co]Cbl or [^99m^Tc]PAMA(4)-Cbl for 20 min (*E. coli*) or 2 h (*S. aureus*) and further exposed to the unlabelled-labelled Cbl mixtures for 10 min (*E. coli*) or 1 h (*S. aureus*).

### Tissue Cage Infection Model in Mice

C57Bl/6 mice from in-house breeding or purchased from Charles River Laboratories GmbH (Sulzfeld, Germany) were housed in the Animal Facility of the Department of Biomedicine, University Hospital, Basel, Switzerland. Experiments were performed in accordance to the regulations of Swiss veterinary law. The Institutional Animal Care and Use Committee approved the study protocol. Drinking water and standard laboratory food pellets (CR) were provided *ad libitum*. To reduce interference of high vitamin B_12_ level in mice, animals randomized for Cbl biodistribution studies were fed with a vitamin B_12_-reduced diet (Provimi Kliba AG, Kaiseraugst, Switzerland) beginning at the age of 10 weeks. At the age of 12 weeks, one sterile polytetrafluoroethylene (Teflon) tube (32 × 10 mm), perforated by 130 regularly spaced holes of 1 mm diameter, was aseptically implanted into the back of each mouse, as previously described [33, 36]. Each cage was weighted and numbered before implantation. Two weeks after surgery, clips were removed from healed wounds, and sterility of the cage was confirmed by culture of aspirated cage fluid. On the following day, 5 × 10^5^ CFU of *E. coli* or 5 × 10^6^ CFU of *S. aureus*, resuspended in 200 μl of 0.9 % saline, were injected into cages. Sterile 0.9 % saline was injected in cages in animals used as negative controls.

### Biodistribution Studies

Biodistribution studies were performed in control and infected mice, 24 h (for *E. coli*) or 48 h (for *S. aureus*) after infection. The bacterial counts in cage fluid were enumerated by plating 10-fold serial dilutions of cage fluid, after 24 h of incubation at 37 °C.

A hundred microlitres of isotonic saline solution containing ≈6 ng/3.3 pmol/10 MBq of [^99m^Tc]PAMA(4)-Cbl and 0.25 mg/≈10 MBq of [^99m^Tc]DTPA was injected into the lateral tail vein of each mouse, randomized for a minimum of nine mice per infection group (*E. coli*, *S. aureus* or sterile mice) per radiopharmaceutical. Distribution of radiopharmaceuticals into the cage fluids was determined at 30 min, 1, 2, 4, 8, 12 and 24 h. Distribution into organs, tissues and explanted cages was measured at 30 min, 4 and 24 h after injection.

One hundred microlitres of isotonic saline solution containing 1.2–2.3 ng/0.9–1.7 pmol /0.014 MBq [^57^Co]Cbl or 0.83 pg/ 0.172 MBq of [^67^Ga]citrate was injected into the lateral tail vein of each mouse, randomized for a minimum of four mice per infection group (*E. coli*, *S. aureus* or control mice) per radiopharmaceutical. Cage fluids were measured at 1, 6, 24, 48 and 72 h p.i. Animals were euthanized at 72 h, and the total-body biodistribution was determined.

The two different experimental set-up of biodistribution were adapted to the known radiopharmaceutical binding/non-binding to plasma protein, persistence in organs and tissues and half-life of the labelling radioisotope.

Accumulation of radiopharmaceuticals in cage fluids was measured by resuspending 100 μl of aspirated fluid in 1 ml PBS and counted in a gamma counter. The percentage of injected dose (% ID_TCF_/ml) was calculated as measured CPM normalized per 1 ml cage fluid and divided by the CPM_0_ (injected dose). For determination of radiopharmaceutical biodistribution, mice were sacrificed with an intraperitoneal injection of 50–80 μl saline solution of pentothal (100 mg/ml). Blood was collected by cardiac puncture, and mice were perfused with 0.9 % saline for around 5 min. Following, tissues were dissected, weighted and collected into test tubes for γ-counter (blood, heart, liver, spleen, stomach, kidneys, lungs, intestine, muscle, bone and cage). The percentage of injected dose (% ID_tissue_/g) was calculated as CPM associated to each organ divided by its weight in grams and by the CPM_0_.

### Statistical Analysis

Comparisons of *in vitro* binding results and *in vivo* biodistribution data were performed using the Student’s *t* test for continuous variables. All results were given as mean values ± SEM, unless otherwise indicated. *P* values of <0.05 were considered significant. All calculations were performed using Prism 4.0a (GraphPad Software, La Jolla, CA, USA).

## Results

### *In Vitro* Binding Studies

**Fig. 3.**
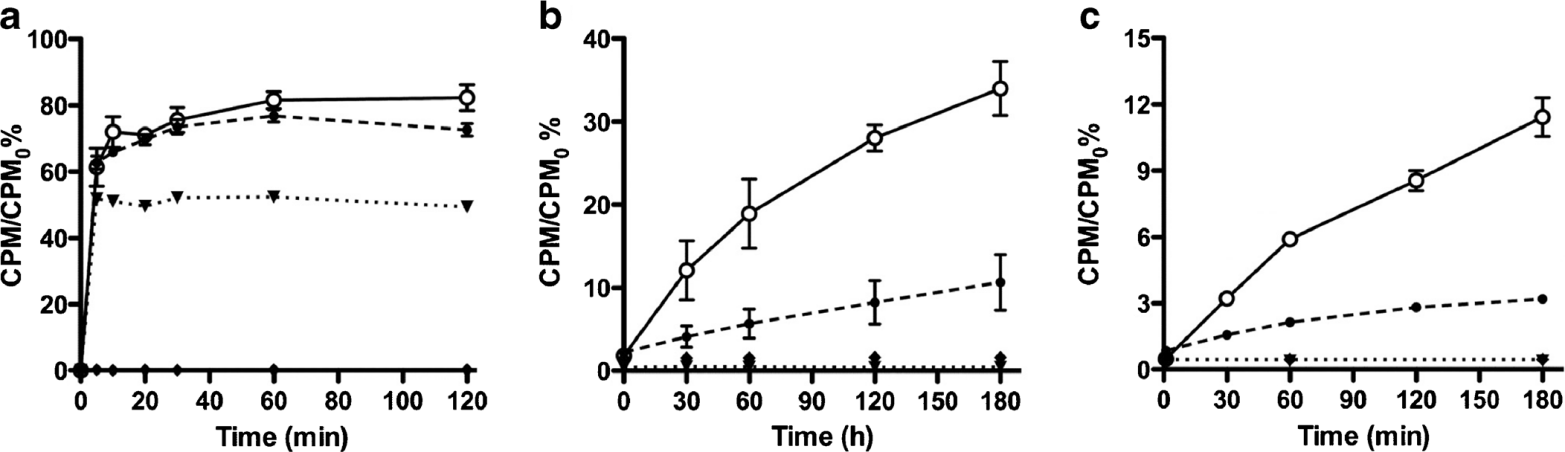
Kinetic of *in vitro* binding (mean CPM/CPM_0_% ± SD) of [^57^Co]Cbl to **a** *E. coli* and **b** *S. aureus* at different incubation times. Kinetic of *in vitro* binding (mean CPM/CPM_0_% ± SD) of [^99m^Tc]PAMA(4)-Cbl to **c** *S. aureus* at different incubation times. At 37 °C (*closed circles*, *continuous line*), 4 °C (*open circles*, *dashed line*), ethanol-killed bacteria (*closed triangles*, *dotted line*) and heat-killed bacteria (*closed diamonds*, *dashed-dotted line*). Note that *X*- and *Y*-axes are scaled depending on the bacterium or radiopharmaceutical tested.

Binding of [^57^Co]Cbl to viable *E. coli* and *S. aureus* was time dependent. *E. coli* showed a rapid binding with plateau reached already 10 min after incubation in a temperature-independent fashion (Fig. 3a). With *S. aureus*, [^57^Co]Cbl displayed a slower binding kinetic and no plateau was reached even after 3 h of incubation (Fig. 3b). In contrast to *E. coli*, binding of *S. aureus* was temperature dependent (approximately 3-fold higher at 37 than at 4 °C). The maximum binding (mean ± SD) was measured at 37 °C and was 81.7 ± 2.6 % for *E. coli* and 34.0 ± 6.7 % for *S. aureus*. Binding of [^57^Co]Cbl to ethanol-killed *E. coli* was lower than the one observed in viable bacteria, while no binding was measured with heat-killed *E. coli*, whereas ^5^[^57^Co]Cbl did not show any binding to both ethanol-killed and heat-killed *S. aureus*.

Binding of [^99m^Tc]PAMA(4)-Cbl to *E. coli* was low, non-displaceable and ranged between 0.1 and 0.3 % in all the tested conditions (living bacteria as well as ethanol- and heat-killed bacteria, data not shown). When testing the *S. aureus* strain, the binding of [^99m^Tc]PAMA(4)-Cbl to viable bacteria at 37 °C and 3 h of incubation was 11.43 ± 1.7 %, and, similarly to the one obtained with the [^57^Co]Cbl, it was slow and temperature dependent, with no binding to killed bacteria (Fig. 3c).

**Fig. 4.**
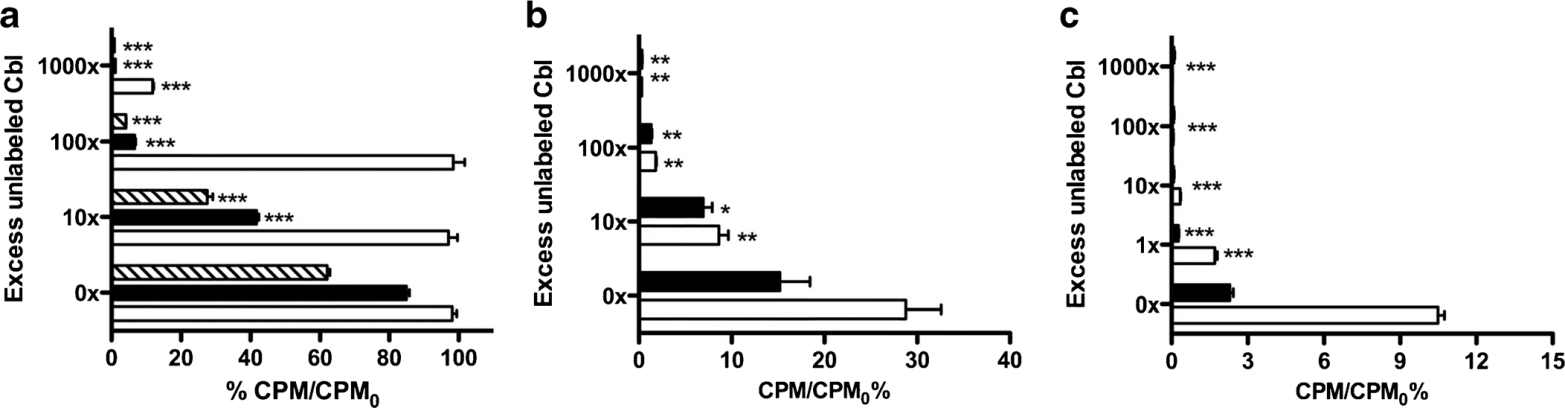
*In vitro* displacement of binding of [^57^Co]Cbl to viable and non-viable **a** *E. coli* and to **b** *S. aureus*; *in vitro* displacement of binding of [^99m^Tc]PAMA(4)-Cbl to **c** *S. aureus*; viable bacteria at 37 °C (*empty bar*) or 4 °C (*filled bar*) and non-viable bacteria after ethanol fixation (*diagonal hatched bars*, *E. coli* only). Significant differences between binding in the absence and in the presence of cold Cbl (at different concentrations) are indicated as follows: *P < 0.05, **P < 0.005, ***P < 0.0005. Note that *X*- and *Y*-axes are scaled depending on the bacterium or radiopharmaceutical tested.

**Fig. 5.**
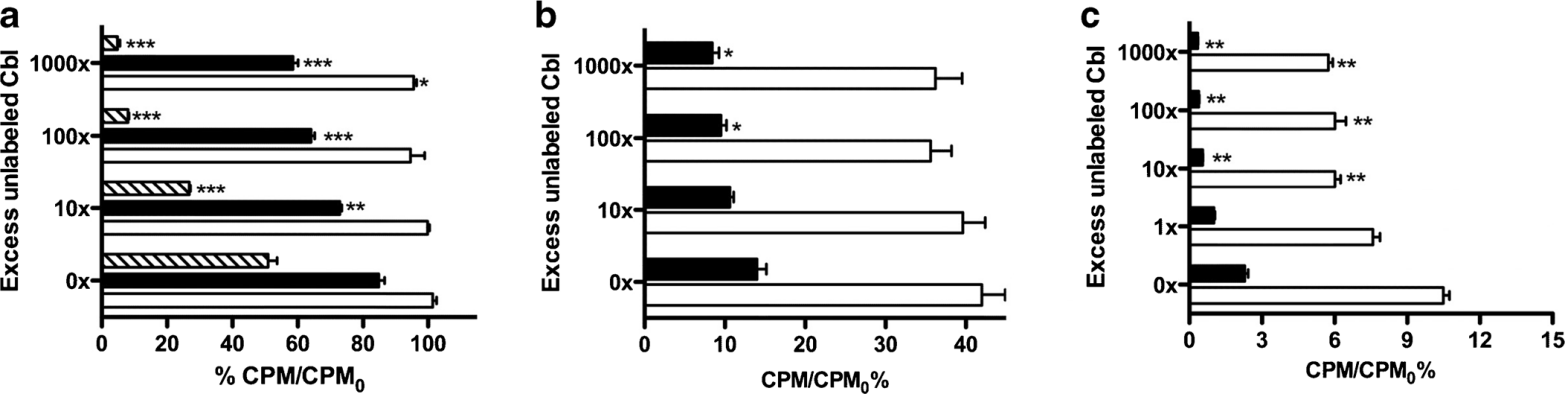
*In vitro* displacement of binding by non-labelled Cbl added after pre-incubation of **a** [^57^Co]Cbl and *E. coli*, **b** [^57^Co]Cbl and *S. aureus* and **c** of [^99m^Tc]PAMA(4)-Cbl and *S. aureus*. Viable bacteria at 37 °C (*empty bar*), at 4 °C (*filled bar*) and non-viable bacteria after ethanol fixation (*diagonal hatched*, *E. coli* only). Significant differences between binding in the absence and in the presence of cold Cbl (at different concentrations) are indicated as follows: *P < 0.05, **P < 0.005, ***P < 0.0005. Note that *X*- and *Y*-axes are scaled depending on the bacterium or radiopharmaceutical tested.

[^57^Co]Cbl could be displaced by non-radioactive Cbl in a concentration-dependent manner. A 1000-fold concentration of unlabeled Cbl was required to show an inhibition of binding to *E. coli* at 37, while at 4 °C and in ethanol-killed bacteria, a 10-fold cold Cbl excess was sufficient to reduce the maximal binding to 50 % (Fig. 4a). The *S. aureus* binding to [^57^Co]Cbl was reduced already by 10-fold excess of unlabeled Cbl at both 37 and 4 °C (Fig. 4b). Already 1-fold of unlabeled Cbl decreased the binding of [^99m^Tc]PAMA(4)-Cbl to *S. aureus* (Fig. 4c). At 37 °C, the binding of [^57^Co]Cbl to *E. coli* achieved after 20 min of incubation was unchanged upon the following addition of the unlabeled Cbl. Differently, at 4 °C, the binding showed a slight decrease when challenged with 1000-fold excess of unlabeled Cbl. Binding of [^57^Co]Cbl to ethanol-killed *E. coli* could be reversed by the excess of cold, independently on the pre-incubation with the labelled Cbl (Fig. 5a). Similar mechanisms were also observed when *S. aureus* was pre-exposed to [^57^Co]Cbl or [^99m^Tc]PAMA(4)-Cbl: the measured binding was only slightly reduced upon later challenge with excess of unlabeled vitamin, both at 37 and 4 °C (Fig. 5b, c).

### Biodistribution Studies

**Fig. 6.**
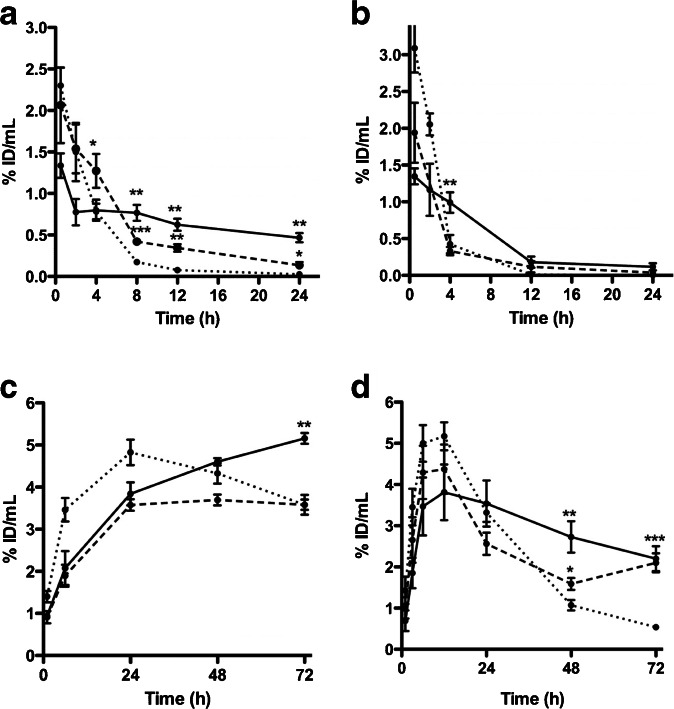
Distribution of **a** [^99m^Tc]PAMA(4)-Cbl, **b** [^99m^Tc]DTPA, **c** [^57^Co]Cbl and **d** [^67^Ga]citrate into tissue cage fluids of sterile (*dotted line*), *S. aureus*-infected (*dashed line*) and *E. coli*-infected (*continuous line*) cages. Data represent % ID/ml of tissue fluid, expressed as means ± 1 SEM of three to five different mice.

On the day of radiopharmaceutical injection, the mean ± SD bacterial counts were 9.4 ± 0.82 log_10_ CFU/ml for *E. coli* and 6.3 ± 0.62 log_10_ CFU/ml for *S. aureus*. Three days after radiopharmaceutical injection ([^57^Co]Cbl and [^67^Ga]citrate biodistribution studies), bacterial counts were 8.6 ± 0.2 log_10_ CFU/ml for *E. coli* and 7.8 ± 0.1 log_10_ CFU/ml for *S. aureus*. Spontaneous cure was not observed in any infected cage. No clinical or pathological signs of systemic infection were observed during organ dissection. The highest concentration of [^99m^Tc]PAMA(4)-Cbl was measured in sterile cage fluids at early time points (Fig. 6a). The maximum [^99m^Tc]PAMA(4)-Cbl % ID_TCF_/ml at 30 min was 2.3 ± 0.39 % for sterile cages, 1.33 ± 0.26 % for infected cages with *E. coli* and 2.06 ± 0.79 % for infected cages with *S. aureus*. Clearance from sterile cages was faster in infected cages, and the % ID_TCF_/ml was significantly lower in controls than in infected mice with *S. aureus* at 4 h (*P* = 0.042) and with *E. coli* at 8 h (*P* = 0.0035). [^99m^Tc]PAMA(4)-Cbl cage fluid/blood ratios discriminated between non-infected mice (1.53 ± 0.30) and infected mice, both with *S. aureus* (13.48 ± 2.75, *P* = 0.0036) or *E. coli* (6.31 ± 2.92, *P* < 0.0001), at 24 h p.i. Indeed, the radiopharmaceutical was rapidly cleared from blood, whereas retention of radioactivity was observed in the kidneys up to 24 h after injection. In explanted cages, the radiopharmaceutical retention did not significantly differ in infected and sterile conditions up to 4 h, but it was discriminative at 24 h for *E. coli*-infected cages (*P* = 0.0375), with % ID/g nearly 10-fold higher than in sterile cages (Table 1).

**Table 1:** Organ and tissue distribution after i.v. injection of [^99m^Tc]PAMA(4)-Cbl, ^[99m^Tc]DTPA, [^57^Co]Cbl and [^67^Ga]citrate in sterile, *E. coli* and *S. aureus* tissue cage infected mice

Organ or tissue	[^99m^Tc]PAMA(4)-Cbl			[^99m^Tc]DTPA			[^57^Co]vitamin B_12_	[^67^Ga]citrate
	30 min	4 h	24 h	30 min	4 h	24 h	72 h	72 h
Blood	1.79 ± 0.05	0.15 ± 0.02	0.02 ± 0.10	1.46 ± 1.10	0.02 ± 0.10	0.01 ± 0.00	2.55 ± 0.35	0.46 ± 0.20
Heart	0.90 ± 0.07	0.33 ± 0.07	0.11 ± 0.01	0.15 ± 1.10	0.05 ± 0.00	0.01 ± 0.00	3.60 ± 0.68	1.26 ± 0.10
Liver	5.75 ± 0.86	2.98 ± 0.45	0.92 ± 0.90	0.33 ± 0.20	0.09 ± 0.00	0.02 ± 0.00	15.78 ± 1.49	7.44 ± 0.90
Stomach	1.73 ± 0.30	0.49 ± 0.23	0.44 ± 0.14	1.67 ± 1.70	0.05 ± 1.10	0.02 ± 0.00	4.83 ± 0.85	3.73 ± 1.50
Spleen	1.48 ± 0.18	0.60 ± 0.07	0.22 ± 0.03	0.19 ± 0.00	0.06 ± 0.00	0.02 ± 0.00	9.07 ± 1.44	6.09 ± 0.70
Kidneys	15.66 ± 1.88	13.47 ± 1.79	11.19 ± 1.35	3.92 ± 0.80	0.93 ± 0.80	0.15 ± 0.00	25.91 ± 4.64	10.73 ± 1.30
Lungs	2.81 ± 0.51	1.09 ± 0.26	0.29 ± 0.10	0.40 ± 0.20	0.06 ± 0.10	0.01 ± 0.00	3.72 ± 1.75	2.11 ± 0.30
Intestine	1.81 ± 0.14	1.52 ± 0.51	0.59 ± 0.05	0.90 ± 0.50	0.66 ± 0.20	0.02 ± 0.00	3.63 ± 0.89	2.00 ± 0.40
Bone	0.74 ± 0.05	0.22 ± 0.01	0.08 ± 0.01	0.47 ± 0.30	0.05 ± 1.50	0.01 ± 0.00	3.93 ± 0.96	10.99 ± 2.40
Muscle	0.38 ± 0.03	0.09 ± 0.01	0.03 ± 0.01	0.34 ± 0.30	0.06 ± 1.10	0.00 ± 0.00	1.42 ± 0.20	0.69 ± 0.20
Cage—sterile	2.37 ± 0.16*	0.87 ± 0.25	0.04 ± 0.01^#^*	2.88 ± 0.50	0.34 ± 0.40*	0.01 ± 0.00	4.60 ± 0.41*	1.45 ± 0.50^#^*
Cage—*S. aureus*	1.80 ± 0.91	1.47 ± 0.38	0.14 ± 0.06	n.d.	0.40 ± 0.10	0.05 ± 0.10	4.88 ± 0.55	9.45 ± 2.30
Cage—*E. coli*	0.76 ± 0.21	0.84 ± 0.23	0.37 ± 0.18	n.d.	0.91 ± 0.15	0.15 ± 0.10	6.52 ± 0.17	3.99 ± 1.10

Values are expressed as mean % ID/g tissue ± SD

*n.d.* not determined

^#^ *P* < 0.04 and 0.004 vs *S. aureus*; *P < 0.005 or 0.03 or 0.003 or 0.001 or 0.02 vs *E. coli*

[^99m^Tc]DTPA was used *in vivo* to evaluate the vascularization of sterile and infected cage fluids. The radiopharmaceutical showed an early peak in cage fluids and cage tissues, ranging between 1.5 and 3 %. Following, clearance was fast from all, sterile and infected cages and tissues (Fig. 6b).

The distribution profile of [^57^Co]Cbl is shown in Fig. 6c. [^57^Co]Cbl displayed a continuous penetration into cage fluids, with peaks achieved between 24 and 48 h in sterile and *S. aureus*-infected cages. A plateau % ID_TCF_/ml of [^57^Co]Cbl into *E. coli*-infected cages was not achieved up to 72 h. Clearance from cage fluids was also slow and was similar in sterile and *S. aureus*-infected mice. Only cages infected with *E. coli* at 72 h showed significantly higher radiopharmaceutical retention in the cage fluid, when compared to control mice (*P* = 0.0032). The cage fluid/blood ratios at 72 h in control mice (1.40 ± 0.19) were slightly discriminative for infected cages with *E. coli* (1.72 ± 0.07, *P* = 0.04), but not with *S. aureus* (1.52 ± 0.10, *P* > 0.05). At 72 h, [^57^Co]Cbl remained in high percentage in the blood, liver and kidneys.

Similarly to [^57^Co]Cbl, [^67^Ga]citrate penetration into cages was gradual, and peak values were achieved within 12 h. Clearance was faster in sterile compared to infected cages and tissues (Fig. 6d). The [^67^Ga]citrate retention in cage fluid became significantly higher in infected than in non-infected animals at 48 h (*P* = 0.029) and 72 h (*P* = 0.0006). At 72 h, accumulation became also significantly higher in explanted cages of infected mice with both *S. aureus* (*P* < 0.0001) and *E. coli* (*P* = 0.0019). The cage fluid/blood ratio at 72 h of [^67^Ga]citrate in non-infected animals was 1.14 ± 0.21, which was lower than in mice infected with *E. coli* (5.41 ± 0.52, *P* < 0.0001) or *S. aureus* (3.56 ± 0.87, *P* = 0.0003). The distribution in tissues and organs at 72 h after injection showed the highest Ga-67 uptake into the liver, kidneys and bone, while the radiopharmaceutical was mostly cleared from the remaining tissues.

## Discussion

*In vitro* studies demonstrated that the bacterial binding of [^57^Co]Cbl is specific and displaceable by excess of unlabelled Cbl. Higher binding avidity was measured for the *E. coli*, rather than the *S. aureus* strain. When *E. coli* and *S. aureus* cultures were pre-exposed to [^57^Co]Cbl, only small fractions of the bound agent could be displaced by unlabeled Cbl, indicating fast internalization. In agreement with an energy-dependent uptake mechanism, the internalized fractions were higher at 37 than at 4 °C, and they were exclusively measured in viable bacteria.

The *in vitro* binding of [^99m^Tc]PAMA(4)-Cbl derivative to *E. coli* was significantly lower for [^57^Co]Cbl. Indeed, the technetium chelator of [^99m^Tc]PAMA(4)-Cbl is linked to the b-acid of the corrin ring A, a functional group involved in hydrogen bonds with the amine groups of Leucin and Alanin residues in the BtuB binding pocket, the *E. coli* outer membrane transporter [18]. In contrast, binding of [^99m^Tc]PAMA(4)-Cbl to *S. aureus* was specific, and only slightly lower than [^57^Co]Cbl. The *S. aureus* receptor mediating Cbl uptake is evidently less affected by Cbl modifications than the *E. coli* outer membrane transporter BtuB.

In animal studies, [^99m^Tc]PAMA(4)-Cbl showed a fast penetration into all cages, followed by a slower release in infected cages than in sterile ones. The retention of this radiopharmaceutical into infected fluids became significantly higher at 4 h p.i. for *S. aureus*-infected mice and at 8 h p.i. for *E. coli*-infected mice, which is in accordance with a lower receptor affinity of PAMA(4)-Cbl for *E. coli* BtuB receptors. The uptake measured in cage fluid of infected animals was also significantly higher for [^99m^Tc]PAMA(4)-Cbl than for the non-specific radiopharmaceutical [^99m^Tc]DTPA. This finding supports a specific interaction of [^99m^Tc]PAMA(4)-Cbl with the colonizing bacteria, rather than a non-specific retention due to the morphological differences between infected and sterile cage fluids.

Differently, [^57^Co]Cbl and by [^67^Ga]citrate had a slow kinetic of penetration into both infected and sterile cages, explained by their high plasma protein binding and long persistence in blood and organs [37, 38]. [^57^Co]Cbl was only partially cleared from sterile cages even after 72 h p.i. Significantly higher retention in sterile mice could be observed in cages infected with *E. coli* at 72 h. The latter result is in accordance to the higher [^57^Co]Cbl binding measured *in vitro* to *E. coli* than to *S. aureus*.

The mechanism of the [^67^Ga]citrate accumulation at infection/inflammation site is associated to the Ga (III) binding to transferrin, lactoferrin and other inflammatory proteins in inflamed sites, internalization into the cells with active metabolic pathway as citrate for citric acid cycle and presumable binding to bacterial siderophores [37, 39]. In our studies, [^67^Ga]citrate discriminated between infected and sterile cages from 48 h after injection. The retention of the radiopharmaceutical observed in the bone, kidneys, liver and spleen is in accordance with data from previous publications [40].

In our cage infection studies, radiopharmaceutical accumulation was several times higher in the cage fluid than in the tissue surrounding explanted cages. While explanted cages contain residues of clotted tissue and cage-adherent bacteria in a stationary growth phase, the cage fluid represents the active infection site with replicating bacteria in the planktonic growth phase [33]. Consequently, in the tissue cage mouse model of infection, cage fluids, rather than explanted cages, may represent a better sample for evaluation of radiopharmaceuticals targeting infection.

## Conclusions

In our study, the tissue cage mice model of infection demonstrated to be a valid alternative to other experimental models for screening radiotracers targeting infection, such as osteomyelitis, infectious endocarditis and infection of thigh muscles [41]. The model has the advantages that infection profiles are highly reproducible planktonic bacterial number is easy to measure, as well as radiotracers kinetic at the site of infection. Indeed, cage fluids can be sampled several times during the experiment, thereby avoiding sacrificing animals for each time point.

Furthermore, we showed that radiolabeled Cbl has a specific receptor-mediated binding to *E. coli* and *S. aureus. In vivo*, the [^99m^Tc]PAMA(4)-Cbl derivative discriminated between infected and non-infected cages in the mouse model within 4 to 8 h after radiopharmaceutical injection, and thus may become a selective radiopharmaceutical for targeting infections in humans.
